# Positive self talk journaling intervention to improve psychological well-being among child and adolescents in juvenile

**DOI:** 10.1186/s13034-025-00998-y

**Published:** 2025-12-12

**Authors:** Iyus Yosep, Nita Fitria, Ai Mardhiyah, Rohman Hikmat

**Affiliations:** 1 Department of Mental Health, Faculty of Nursing, Universitas Padjadjaran, Sumedang, Jawa Barat Indonesia; 2 Department of Fundamental Nursing, Faculty of Nursing, Universitas Padjadjaran, Sumedang, West Java 45363 Indonesia; 3 Department of Pediatric Nursing, Faculty of Nursing, Universitas Padjadjaran, Sumedang, West Java 45363 Indonesia; 4 Faculty of Nursing, Prince of Songkla University, Hat Yai District, Songkhla, 90110 Thailand

**Keywords:** Adolescents, Journaling, Positive self talk, Prisoners

## Abstract

**Background:**

Juvenile inmates often face psychological pressures such as stress, anxiety, and depression while serving their sentences. These conditions can reduce their psychological well-being. Previous research has shown that cognitive approaches such as positive self-talk are effective in helping individuals manage negative thoughts, while journaling can improve self-awareness and emotion regulation. However, there have been few interventions that combine these two approaches in juvenile inmates.

**Objective:**

to determine the effect of positive self-talk journaling intervention on the psychological well-being of juvenile prisoners.

**Method:**

this study used a quasi-experimental design with a control and intervention group. The research sample consisted of 110 juvenile prisoners who were selected purposively according to the inclusion criteria, namely aged 13–18 years and willing to participate in the program. Data collection used the Ryff’s Psychological Well-Being Scale at two times, before and after the intervention. The intervention was given for 4 weeks with structured sessions for positive self-talk and journaling. Data analysis was performed using the Wilcoxon test for analysis within groups and the Mann-Whitney test for comparison between groups.

**Results:**

the Wilcoxon test results showed a significant increase in psychological well-being scores after the intervention in the intervention group (*p* < 0.05). The Mann-Whitney test showed a significant difference between the intervention group and the control group after the intervention (*p* < 0.05). These findings indicate that the positive self-talk journaling program is effective in improving the psychological well-being of juvenile prisoners. This intervention has a positive impact by helping juvenile prisoners recognize negative thoughts and replace them with positive affirmations.

**Conclusion:**

journaling techniques allow for deep self-reflection, thereby improving their ability to manage emotions. These findings support empirical evidence that cognitive and emotional-based approaches can improve mental health in high-stress situations.

## Introduction

Psychological well-being is a vital aspect of an individual’s life that involves feelings of emotional security, the ability to manage stress, and positive social relationships [41]. For adolescents, psychological well-being plays a more significant role, because adolescence is a critical period in the formation of self-identity, the development of emotional independence, and the formation of self-confidence [3]. Adolescents who have good psychological well-being tend to be able to overcome challenges more effectively, have a more optimistic outlook on life, and are able to establish healthy social relationships. In addition, good psychological well-being is also important to support the learning process, adaptation skills, and the development of self-potential in adolescence, which is a dynamic and changing stage of development [4].

The psychological well-being of juvenile prisoners is often threatened by severe stress arising from the physical and social situations they face. Life in prison deprives them of freedom, access to the social support they need, and creates various forms of social stigma that damage their self-esteem. In these conditions, juvenile prisoners often experience feelings of isolation, anxiety, and low self-esteem that can worsen their psychological well-being [29]. Limited interaction with family or peers also adds to the emotional stress they feel, reducing opportunities for moral support, and making them more vulnerable to mental disorders. If not addressed with the right approach, this psychological stress can cause long-term problems for their mental and emotional development, so interventions are needed that are specifically designed to improve the psychological well-being of adolescents in stressful environments such as prison [37].

Very high prevalence of psychological problems among juvenile prisoners, with the proportion of anxiety disorders, depression, and low self-esteem far exceeding that of adolescents in the general population. Previous report that around 70–80% of juvenile prisoners experience serious anxiety symptoms, such as intense feelings of fear, chronic restlessness, and an inability to cope with daily stress [21]. In addition, more than 60% of this population experience symptoms of major depression characterized by feelings of hopelessness, loss of motivation, and low energy that hinder their daily activities [13]. Low self-esteem is also found in more than 65% of juvenile prisoners, reflected in feelings of worthlessness, low self-confidence, and reluctance to interact with others [10].

Psychological interventions are critical to the well-being of incarcerated youth who face unique emotional and psychosocial challenges due to the stress of the prison environment, social stigma, and limited support [39]. Adolescents in these settings are vulnerable to mental disorders such as anxiety, depression, and low self-esteem, which can potentially hinder their resocialization. Appropriate interventions not only help them manage their emotions and strengthen their positive identities, but also play a long-term role in increasing their successful reintegration into society and reducing the risk of reoffending [25, 42]. While studies have shown that counseling and group therapy are effective in improving the psychological well-being of incarcerated youth, more innovative techniques, such as journaling or therapeutic writing, have not been widely used [14, 21].

Positive Self Talk is a psychological technique that helps individuals direct their thoughts in a constructive direction through positive internal dialogue, thereby increasing positive thought patterns, reducing negative thoughts, and supporting self-development [8]. This technique works by replacing destructive thoughts with positive messages that can increase motivation and self-confidence. On the other hand, journaling serves as a self-reflection tool that allows one to record and explore their thoughts and feelings on a regular basis, which is effective in emotional management and tracking psychological progress [12]. The combination of Positive Self Talk and journaling provides a positive synergy, by providing a space to instill positive beliefs through Self Talk while exploring and reflecting on emotional experiences through journaling [9, 28].

Previous studies have explored the effects of Positive Self Talk and journaling separately in adolescent populations, suggesting that both can improve psychological well-being by reducing symptoms of anxiety and depression, and increasing self-esteem [1, 31]. However, there is a significant gap in the literature, namely the lack of research examining the combined effects of Positive Self Talk and journaling in adolescent prisoners, a group that faces unique emotional and psychosocial challenges. The combination of these two techniques is relevant and potentially effective in improving the psychological well-being of adolescent prisoners because it can provide a comprehensive coping strategy [2, 19, 27, 44]. Positive Self Talk helps them overcome stigma and limitations in the prison environment, while journaling allows exploration and reflection on their experiences and emotions. By combining these two approaches, adolescent prisoners can better manage stress, increase self-esteem, and develop reflective skills that support their future resocialization process. The purpose of this study was to determine the effect of positive self-talk and journaling techniques in improving psychological well-being in adolescent prisoners.

## Methods

### Study design

This study used a quasi-experimental design with a pre-test and post-test and a control group. This design was chosen because it allowed researchers to evaluate the effects of the intervention without full randomization, which is often impractical in prison settings. By having a control group, researchers were able to compare changes in psychological well-being between groups that received the intervention and those that did not. The intervention group underwent a positive self-talk journaling program for 3 months, while the control group continued their normal routines without the intervention. This treatment ensured that any differences in outcomes observed could be more reliably attributed to the intervention.

### Participants

Participants in this study were juvenile prisoners at Special Development Institution (LPKA) in Bandung City. The inclusion criteria set were: (1) prisoners who would be inmates for at least the next 3 months, and (2) able to communicate well in Indonesian. These inclusion criteria were chosen to ensure that participants could follow the program well and had enough time to experience the effects of the intervention. Meanwhile, the exclusion criteria included: (1) prisoners who were not in LPKA for the next 3 months, and (2) prisoners who had mental retardation, which was feared to affect their ability to follow the program and understand the instructions given.

The sampling technique used in this study was total sampling, as all juvenile inmates who met the eligibility criteria during the study period voluntarily agreed to participate. A total of 110 participants were recruited and randomly assigned into two groups, with 55 individuals in the intervention group and 55 in the control group. The final sample size was determined based on the total number of eligible juvenile inmates available during the study period. Although a formal power analysis was not conducted due to limited access to the correctional population, the sample was considered adequate for detecting meaningful group differences in pre- and post-test outcomes.

### Procedure

#### Overview and attention control

Across the 3-month intervention period, both groups met weekly for approximately 60 min in a private, distraction-free room at LPKA with the same facilitator. To ensure parity of attention and minimize non-specific effects of facilitator contact and social interaction, the control group attended eight neutral discussion sessions (e.g., hobbies, sports, prison activities) that intentionally excluded journaling and any psychological skills training. The intervention group attended eight positive self-talk journaling (PSTJ) sessions as described below.

- Pre-experimental measurement and group assignment

Recruitment was conducted by a research assistant who disseminated informed consent and screened candidates against inclusion/exclusion criteria. Consented, eligible participants completed the Ryff’s Psychological Well-Being Scale (PWBS) as the baseline assessment (pre-test). Participants were then randomly assigned to the intervention or control group using a computerized randomization tool (https://www.randomizer.org). While blinding of participants/facilitator was not feasible due to the nature of the activities, the data analyst was blinded to group assignment to reduce interpretive bias.

b. Implementation of sessions

The PSTJ program consisted of 8 weekly sessions (~ 60 min each) delivered by a trained facilitator with a mental health background. Sessions combined check-ins, psychoeducation, skills training, group interaction, and individual journaling; participants submitted journals confidentially after each session and received brief written feedback (Table 1).

**Table 1:** Structure and content of the PSTJ sessions

Session	Content of positive self-talk journaling (PSTJ)
1	Introduction to self-talk and journaling; distinguish positive vs. negative self-talk; reflective writing on a time negative self-talk affected actions or self-view
2	Techniques for positive self-talk: affirmations and visualization; create personal affirmations; practice visualization; journal a plan for use during the week
3	Reflection and refinement; share experiences applying techniques; troubleshoot barriers; journal changes in emotions, thoughts, or behavior
4	Building consistency; habit strategies (reminders, pairing with routines, simple log); journal how self-talk was integrated into daily life
5	Managing setbacks; identify warning signs and recover from dips; problem-solve real cases; journal a recent challenge and an adaptive response
6	Self-compassion; guided reflection on forgiveness and self-wort

Each session began with a check-in, followed by psychoeducation, skills training, group interaction, and individual journaling. Participants submitted their journals confidentially after each session, and researchers provided brief written feedback.

#### Control (attention-matched neutral discussions)

In parallel with the intervention schedule, control participants attended 8 weekly ~ 60-min sessions facilitated by the same person, covering neutral, non-therapeutic topics (e.g., hobbies, sports, routine prison activities). No journaling, psychoeducation, or psychological skill-building occurred in these sessions.

c. Post-experimental measurement and closure

At the end of the 3-month period, both groups completed the PWBS post-test. Participants then received a certificate of appreciation, with acknowledgment for the most engaged attendees. The institution received a token of gratitude and a formal report on study outcomes.

### Data collection

The researcher used the Indonesian-translated and validated version of the Ryff’s Psychological Well-Being Scale (PWBS), consisting of 17 items. This version has undergone psychometric testing in previous studies. Construct validity was demonstrated through item-total correlation values above 0.30 and factor loadings above 0.50 for all items. The internal consistency of the Indonesian adaptation of the Psychological Well-Being Scale was supported by strong reliability coefficients with Cronbach’s alpha was 0.809 and ranged from 0.543 to 0.828 [6, 11]. Measurement of psychological well-being was interpreted as follows: low (< 40), medium (40–60), and high (>60), which allows for in-depth analysis of the psychological well-being levels of participants.

### Data analysis

Data analysis will be conducted using univariate and bivariate tests using SPSS version 28. Univariate analysis aims to describe demographic characteristics and research variables by presenting the mean, minimum, maximum, and standard deviation values ​​in tabular form. To test the hypothesis, the Wilcoxon signed-rank test will be used to analyze the differences in the intervention group before and after the intervention. Meanwhile, to compare the effects of Positive Self Talk Journaling between the intervention and control groups, the Mann-Whitney test will be applied because the data is not normally distributed.

### Ethical considerations

This study has obtained ethical clearance from the Research Ethics Committee of Universitas Padjadjaran, Bandung with number No. 1079/UN6.KEP/EC/2024, which reflects a commitment to protecting the rights and welfare of participants. The principle of autonomy is applied by providing participants with the opportunity to provide informed consent, while beneficence ensures that the research is designed to provide benefits to participants. Maleficence is prioritized with steps to minimize risks, and veracity is upheld through the delivery of accurate and transparent information. The selection of participants was carried out fairly according to the principle of justice, and the confidentiality of participant data was strictly maintained to protect their identities and personal information by only storing data for 2 years.

## Results

### Baseline characteristics

At pre-test, the two groups were comparable. Age did not differ significantly between the intervention and control groups (independent-samples t-test, *p* = 0.099; overall mean ≈ 15.37 years), and most participants were 13–19 years old (70.9%). Education levels were similarly distributed across groups [elementary 29.1%, junior high 34.5%, senior high 36.4%; chi-square *p* = 0.596). Incarceration duration was also comparable (≥ 1 year: 56.4% in the intervention group and 52.7% in the control group; chi-square *p* = 0.698)]. A total of 110 adolescents contributed analyzable data (intervention = 55; control = 55); all completed both pre- and post-tests.

### Primary outcome (PWBS)

**Fig. 1:**
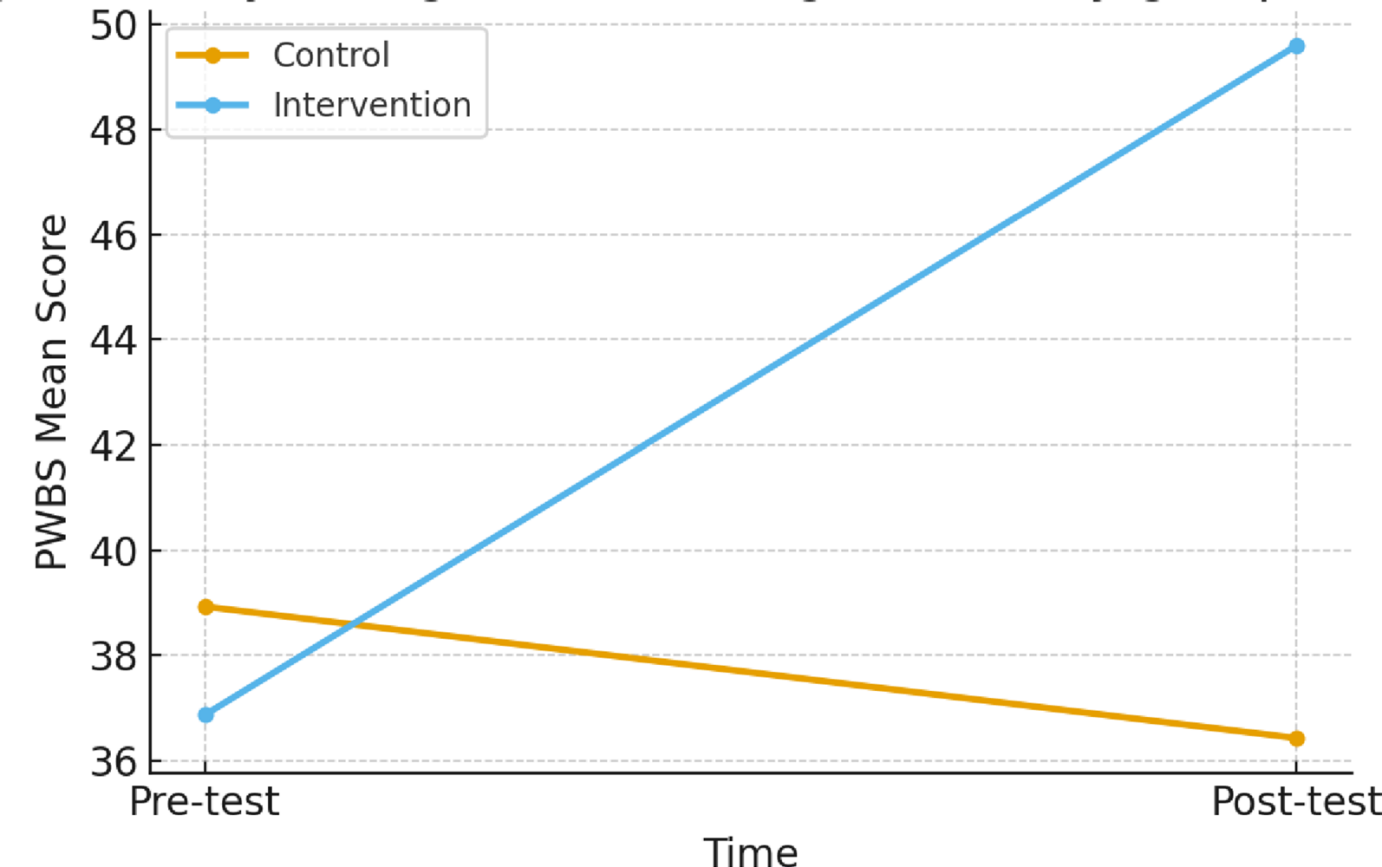
Psychological well-being (PWBS) by group over time (95% CI)

The primary analysis used a 2 × 2 repeated-measures ANOVA (Group: intervention vs. control; Time: pre vs. post). Results indicated a significant main effect of time, F(1,106) = 34.21, *p* < 0.001; a significant main effect of group, F(1,106) = 6.01, *p* = 0.016; and a significant Time-by-Group interaction, F(1,106) = 42.28, *p* < 0.001 (see Fig. 1; Table 2). These findings show that psychological well-being increased substantially in the intervention group but not in the control group.

**Table 2:** Estimated means (95% CI) and between-group difference in change (PWBS)

Time	Intervention mean (95% CI)	Control mean (95% CI)	Between-group difference in change (post–pre)	*p*-value
Pre-test	36.87 (34.64–39.10)	38.93 (36.32–41.54)	NA	NA
Post-test	49.60 (47.00–52.20)	36.43 (33.72–39.14)	**+ 15.23**	**< 0.001**

Note. 95% confidence intervals are based on group standard deviations and *n* = 55 per group. The p-value refers to the Time-by-Group interaction from the repeated-measures ANOVA; nonparametric tests (Wilcoxon within-group; Mann–Whitney between-group) show the same pattern

*NA* Not applicable

### Observed means (95% CI)

At pre-test, mean PWBS was 36.87 (95% CI 34.64–39.10) in the intervention group and 38.93 (95% CI 36.32–41.54) in the control group. By post-test, the intervention increased to 49.60 (95% CI 47.00–52.20) whereas the control declined to 36.43 (95% CI 33.72–39.14), yielding a between-group difference in change of + 15.23 points (*p* < 0.001; see Table 2; Fig. 1).

### Supporting analyses

Within-group change was significant for the intervention (Wilcoxon Z = − 6.397, *p* < 0.001; mean change + 12.73), but not for the control group (Z = − 1.476, *p* = 0.140; mean change − 2.50). A between-group comparison at post-test also supported the ANOVA (Mann–Whitney U = 423.5, Z = − 6.539, *p* < 0.001). Together with the significant Time-by-Group interaction, these results indicate a moderate-to-large intervention effect.

### Participant flow and baseline comparability

All eight sessions were delivered as scheduled. Both groups provided complete pre- and post-test data (*n* = 55 per group). Baseline characteristics were comparable: age (*p* = 0.099), current education (*p* = 0.596), and incarceration duration (*p* = 0.698); gender distribution also did not differ significantly between groups (chi-square, *p* > 0.05). These checks are intentionally concise to document comparability while keeping the focus on longitudinal effects.

## Discussion

The purpose of this study was to determine the effect of a positive self-talk journaling intervention on the psychological well-being of juvenile prisoners. In line with this objective, the analysis showed that the intervention group experienced a clear increase from pretest to posttest, while the control group did not improve and tended to decline. The effect of time by group was significant, indicating that the intervention was superior to the matched control. This core finding represents the study’s primary contribution, demonstrating the benefits of a structured journaling protocol for a population of adolescents in a stressful correctional environment.

Journaling-based interventions that focus on positive self-talk provide benefits in several aspects. First, journaling activities allow adolescents to externalize negative thoughts, identify destructive thought patterns, and practice self-affirmations that can improve their self-image [5]. Thus, this program can effectively reduce the level of anxiety, feelings of hopelessness, and low self-esteem that are often experienced by adolescents during the correctional period. Psychological programs designed to help inmates cope with their emotional distress play a major role in achieving positive rehabilitative outcomes and reducing the risk of reoffending [7].

These findings are consistent with the cognitive restructuring framework, which states that improvements in emotional functioning occur when maladaptive thoughts are recognized and replaced with more constructive appraisals through reflective practice and self-reflection, as demonstrated in young populations in cognitive intervention and reflective writing studies [17, 23]. This mechanism is also consistent with evidence that self-affirmation and reappraisal practice are associated with improvements in well-being indicators, including self-acceptance and personal growth relevant to the Psychological Well-Being Scale [18].

The strength of the intervention group’s effects likely stems from the tiered curriculum design, which progresses from mindful self-talk, affirmations, visualization, daily practice, self-compassion, and identity reconstruction. This gradual structure encourages cognitive rehearsal and habit formation, making adaptive statements more easily internalized [32]. The emphasis on self-compassion and self-reconceptualization helps neutralize threat-based self-schemas while enhancing a sense of agency, which is particularly relevant for adolescents in restrictive environments [32, 43]. Furthermore, confidential journaling with brief written feedback provides a safe space for labeling emotions, organizing experiences, and gradual reflection, facilitating emotional reappraisal and regulation [26, 30].

The context of adolescent development reinforces the importance of positive self-talk journaling. Adolescence is characterized by the search for identity and autonomy, which increases vulnerability to psychosocial stress, particularly in environments with restrictions on freedom, as described in the developmental and juvenile justice literature [22]. Accordingly, training in independently practicable cognitive skills, such as positive self-talk, aligns with adolescents’ self-regulation needs and the maturation of their executive functions, which are still developing [24]. Implementation through structured journaling allows for repeated practice, metacognitive reflection, and internalization of adaptive statements, all of which support improved psychological well-being in young populations [40].

Institutional characteristics help explain differences in trajectories between groups: incarcerated youth are exposed to chronic stressors and separation from family support, which are consistently associated with decreased well-being over time [20, 34]. Without skills training, mindfulness and social contact alone are typically insufficient to prevent psychological deterioration, consistent with the small decline in the control group in this study [7, 16]. In contrast, improvements in the intervention group are consistent with cognitive restructuring mechanisms, where guided self-talk helps identify and replace maladaptive thoughts with more adaptive appraisals [17, 23]. The structured journaling component also provides a safe medium for emotional labeling and reappraisal, supporting emotion regulation under high-stress conditions [27, 30].

The acceptability of the intervention is also related to the educational background of the participants. Differences in literacy skills and understanding of instruction directly impact the quality of reflective writing and the independent application of cognitive strategies, making scaffolding through concrete examples, step-by-step instructions, and explanations of technical terms crucial to ensuring implementation in adolescents [33, 35]. In this program, the concise and repetitive session design, short assignments, and written feedback at the end of sessions likely reduced cognitive load and bridged the literacy gap often reported in school contexts [12]. This stepwise approach aligns with findings that repeated practice through journaling strengthens the automation of emotion regulation strategies and facilitates the transfer of skills beyond formal sessions [30].

The matched-attention control design strengthens internal validity by minimizing the likelihood that improvements are driven solely by facilitator contact or group dynamics, rather than by the skills taught. The standardization of the eight-session curriculum and blinding of data analysts to group assignments contribute to fidelity of implementation and reduce interpretation bias. Furthermore, structured journaling practices have been reported to be acceptable and beneficial for at-risk youth and in high-stress settings, supporting the applicability of these findings to similar field contexts [7, 17, 38, 41]. The consistency of the pattern of findings between the parametric and nonparametric analyses aligns with reports of writing-based cognitive interventions in youth populations, which have demonstrated benefits on indicators of well-being and emotion regulation [15, 23].

### Policy implications

Key findings support the integration of positive self-talk journaling (PSTJ) as a routine module in LPKA due to its low cost, minimal resource requirements, and appropriateness to privacy and consistency of practice. Structured psychological support is associated with better rehabilitative outcomes and reintegration readiness [8, 36]. Implementation can begin with a brief, eight-session curriculum-based facilitator training, fidelity monitoring, and pre- and post-test outcome evaluations on Psychological Well-Being. To maintain safety and ethics, establish basic referral pathways and data protection procedures. The feasibility and scalability of PSTJ are strengthened by evidence that structured journaling is acceptable and beneficial for at-risk youth and high-stress services [38].

### Limitations

Limitations in this study are related to scheduling and implementation time that can affect the accuracy of the results. The scheduling of activities has been agreed upon with the LPKA, but the scheduling process that must be adjusted to other activities at the LPKA, such as visits from the Ministry of Law and Human Rights and internal activities that precede the intervention, causes delays in the implementation of activities. This results in a mismatch between the planned schedule and the actual implementation time, which can affect the effectiveness of the intervention on the psychological well-being of participants.

In addition, this study is at risk of selection bias because it did not use a randomization method in selecting respondents, which could cause the results of the study to be influenced by other uncontrolled factors. Confounding factors, such as daily activities carried out by inmates (e.g. spiritual guidance, sports activities, and arts) and social interactions with fellow inmates in LPKA, can affect the level of psychological well-being and intervention outcomes. The presence of these external factors can cause unexplained differences between the experimental and control groups.

## Conclusion

This study indicates that positive self-talk journaling is a feasible and impactful approach to strengthening psychological well-being among adolescents in custody. By combining cognitive restructuring, reflective writing, and self-compassion, the intervention helps shift internal dialogue toward more adaptive patterns. These features make it well suited to high-stress institutional contexts and clarify the study’s central contribution.

In practical terms, the program is low cost, straightforward to integrate into existing schedules, and deliverable by trained facilitators with brief supervision. A phased adoption within LPKA can be considered as part of routine psychological support. Basic implementation monitoring and outcome tracking would help ensure quality and sustainability. Nurses working with juvenile inmates in correctional facilities can utilize Positive Self Talk and journaling techniques as part of a therapeutic approach to improve the psychological well-being of inmates. Nurses can help inmates develop skills in positive thinking and self-reflection, which are important in supporting their rehabilitation process. Future studies should explore the long-term effects of Positive Self Talk and journaling techniques on the psychological well-being of juvenile inmates, as well as their impact on their behavior in the long term by reducing the risk of bias by using a randomized control trial design.

## Data Availability

The data that support the findings of this study are available from the corresponding author, [IY], upon reasonable request.
